# Basel III FRTB: data pooling innovation to lower capital charges

**DOI:** 10.1186/s40854-021-00252-2

**Published:** 2021-05-13

**Authors:** Jimmy Yicheng Huang

**Affiliations:** McGill University, Montreal, Canada

## Abstract

**Background:**

Anticipated to overhaul the structure of market risk teams, IT teams, and trading desks within banks by 2023, Basel III's Fundamental Review of the Trading Book requirements will also increase capital charges banks will incur globally. The case study focuses on describing what is needed with regards to the risk factor eligibility test (RFET) as well as for implementing a data pool to lower capital charges. By establishing a consortium of banks per region to implement a data pooling solution, participants can prove a wider breadth of modellable risk factors per asset class and use the Internal Models Approach (IMA) of valuing risk to lower capital charge requirements significantly.

**Case description:**

First, a description on the historical context surrounding the Fundamental Review of the Trading Book rules and the business requirements needed to comply with the risk factor eligibility test is made. Then an examination is conducted on the innovative data pooling initiative implemented by CanDeal, TickSmith Corp., and the 6 largest Canadian banks to lower capital charge requirements under the Fundamental Review of the Trading Book.

**Discussion and evaluation:**

A description is made on what types of data, expertise, and technology is needed to calculate for risk factor modellability. It is up to each firm to decide if the benefits to using the Internal Models Approach to lower capital charges outweighs implementation and running costs of the underlying data platform. Implementing a data pool for each region comes with challenges that include anti-competition law that may block the initiative, varied benefits to each competitive participant, and data security concerns.

**Conclusion:**

It is evident that the data pool innovation provides benefits to lowering capital charges as the Canadian banks have seen an increase of modellability by several factors using the sample bond asset class. While each firm must still determine internally if the benefits outweighs the technological costs they will incur, it is clear that regulators are pushing for increased data retention and scrutiny.

## Introduction to data pooling for Basel III FRTB

The paper provides a description on the technological and business-related requirements needed for firms to lower capital charges under the Fundamental Review of the Trading Book (FRTB) “Minimum Capital Requirements for Market Risk” component of the Basel III framework specifically with regards to meeting the risk factor eligibility test (RFET). Additionally, a novel method is proposed to lower capital charges under FRTB modellability rules through implementing a data pooling service by establishing a consortium of banks and dealers per region. As of this paper’s publication, Canada is the only country that has successfully implemented a data pooling service using country-wide OTC price observation data for FRTB and there has been no other academic research published on how data pooling, from a bank consortium’s perspective, captures usable data points to lower capital charges. The paper will provide insights on the efficacy of data pooling to lower capital charges by taking metrics from the BCBS *Quantitative Impact Study* and comparing that with modellability results from the Canadian data pooling solution aggregating OTC trade data from the following participants: *BMO Nesbitt Burns Inc., CIBC World Markets, National Bank Financial Inc., RBC Capital Markets, Scotia Capital*, and *TD Securities*.^1^

The paper conducts a macro-level examination on the nature of FRTB modellability while also serving as a useful guide in navigating both the increasing computational complexities as well as data retention requirements within the modern data-focused regulatory climate with frameworks such as Basel III, Solvency II, and MiFID II among others. The technological capabilities of a data pool to ingest, aggregate, and anonymize underlying datasets can be a useful solution to other regulations with parallels to Basel III FRTB such as the Solvency II framework where insurance firms must similarly cover financial resources by supervising and reporting on risk assessments. Note that while the finalized FRTB rules have been published January 14 2019, the dynamic nature of regulations such as these may render details within the paper outdated in the coming years. Regardless of changes to regulations, the general guideline on how firms can benefit from technological innovation in regulatory compliance will still hold.

## International market risk updates post-2007 financial crisis

The 2007–08 financial crisis occurred in large part as a result of complex packaged and tranched securities such as collateralized debt obligations being both mispriced and misrated by financial institutions. A multitude of factors contributed to how these bundled securities became misrated, these factors include, but are not limited to, the irresponsible underwriting of mortgage-related securities,^2^ alleged misrepresentation by rating agencies of the risks associated with these securities,^3^ and the general belief within the United States that mortgage-related debt were safer investments than they were in actuality.^4^ In the mid-2000s, American banks as well as many other international banking institutions lacked both a rigorous methodology to accurately price certain illiquid securities as well as sufficient capital charge requirements to weather the default of these securities.

Basel III is an international framework developed by the Basel Committee on Banking Supervision updated in response to the financial crisis of 2007–08 to enforce a stricter methodology to track continuous prices and notional amounts from securities as well as to increase associated capital charge requirements. Other priorities the framework tackles include strengthening the regulation, supervision, and risk management of banks. As of March 2019, all 27 member jurisdictions including Canada, the European Union, China, and the United States have enforced relevant risk-based capital rules.^5^ 21 member jurisdictions have issued final rules for the revised securitisation framework.^6^

Additionally, the FRTB set of rules published by the Basel Committee introduces new regulations to valuing risk and market positions. The pre-crisis approach assumed that aggregating a portfolio of securities lowers exposure to different risks, however, the fact that a host of bundled mortgage securities defaulted during the 2007–08 financial crisis showed that combining mostly low quality assets should not necessarily enhance their valuation in the way it had been previously calculated to.^7^

Originally, the finalized 2019 FRTB framework outlined that each trading institution that participated in the markets should integrate the new FRTB rules into their security pricing methodology by January 1 2022.^8^ However, as a result of disruptions from the COVID-19 pandemic on the world economy in 2020, this implementation date has been delayed by the *Basel Committee's* oversight body, the *Group of Central Bank Governors and Heads of Supervision (GHOS),* to a January 1, 2023 implementation date.^9^

## Internal models approach and standardized approach literature review

Under the Basel III FRTB rules, all trading desks must calculate minimum capital risk requirements using the Standardized Approach (STA),^10^ however, a less capital-intensive calculation option exists called the Internal Models Approach (IMA) that may apply to certain securities and desks. A series of steps are involved for the approval to calculate the minimum capital risk requirements per trading desk under the Internal Models Approach (IMA). The steps involved are to nominate a trading desk, pass an overall assessment by a supervisor, and lastly to prove the frequency of observable prices over history per risk factor traded.^11^ The methodology outlined in FRTB to prove the frequency of observable prices is also known as the risk factor eligibility test (RFET).^12^

Having the option to use IMA for assessment gives banks the opportunity to lower capital charges on their trading book. In April 2020, the *Basel Committee on Banking Supervision’s (BCBS) Quantitative Impact Study (QIS)* published percentage breakdowns of minimum capital requirements for market risk by approach and risk component in the statistical annex section of the report.^13^ The respondents that contributed to this overall study include a variety of “Group 1” banks defined as firms having Tier 1 capital of more than €3 billion such as *Deutsche Bank*, *HSBC*, and *JPMorgan Chase*. The conservative estimations among 47 surveyed “Group 1” banks for mean minimum capital requirements under the revised Standardized Approach using the sensitivities-based method is 35.2% whereas the mean minimum capital requirements under the revised Internal Models Approach using modellable risk factors is 19.6%.^14^ 23 of these 47 “Group 1” bank respondents are considered to be global systemically important banks (G-SIBs). In order to use IMA, a firm must have enough real price observation data points to determine the modellability of the risk factors it uses. Essentially, the difference between the 35.2% mean capital requirement figure using STA and the 19.6% IMA figure represents the expected percentage of savings in capital charge costs the average “Group 1” bank may achieve by sourcing enough data to produce modellability in the instruments they currently hold or would like to trade.

The official *Minimum capital requirements for market risk* document published by the Basel committee states:

1. The bank must identify for the risk factor at least 24 real price observations per year (measured over the period used to calibrate the current ES model, with no more than one real price observation per day to be included in this count). Moreover, over the previous 12 months there must be no 90-day period in which fewer than four real price observations are identified for the risk factor (with no more than one real price observation per day to be included in this count). The above criteria must be monitored on a monthly basis; or
2. The bank must identify for the risk factor at least 100 “real” price observations over the previous 12 months (with no more than one “real” price observation per day to be included in this count).^15^

Adopting a rigorous methodology to show continuity of prices, such as under the January 2019 FRTB modellability (RFET) requirements, comes at a technological cost in storing and processing data as well as to implement and support the business logic. It is up to each organization to determine whether having lower capital charges from advanced price observation methodology outweighs its implementation and running costs. Additionally, as a result of global technological innovation in the storage and computation of data, it is likely that future regulators in the capital market space will only strengthen data retention requirements therefore the adoption of massively distributed data management capabilities is recommended regardless.

Additional literature surrounding how Standardized and Internal Model approaches may be implemented in anticipation of the FRTB framework coming into effect includes more efficiently calculating downstream sensitivities as detailed by Zhan’s “Calculation of Sensitivities for FRTB Standardized Approach”^16^ and solving computational challenges as explained by Zeron Medina Laris and Ruiz’s “Denting the FRTB IMA Computational Challenge via Orthogonal Chebyshev Sliding Technique”^17^ paper.

In the following section, an examination will be made on the requirements and costs associated with implementing these modellability rules using the example asset class of bonds.

## Technological costs to calculating bond modellability (finalized 2019 FRTB rules)

The technological requirements and costs to calculate modellability can be separated into four categories: implementing business logic, data storage, computation, and continuous information technology support. The costs in all these areas can vary significantly for each institution depending on multiple factors including the size of the firm, budget allocated, operational efficiency, the state of existing legacy systems, and disparities per geography in the wages of employees. To give example budgets using two global systemically important banks, *JPMorgan Chase’s* CFO, Marianne Lake, explained on their Q1 2019 earnings call that *JPMorgan Chase* is spending approximately half of their total $11.5 billion USD technology budget or $5.75 billion USD specifically on technological innovation^18^ while in the same year *HSBC’s* CIO, Darryl West, was quoted that HSBC’s annual spend on technology investments is approximately $3 to $3.5 billion USD.^19^

To implement business logic, explicit costs will come from building regulatory and technological knowledge within the organization and the wages of employees working on calculating modellability. Implicit costs will come from the time firms take to gather data, make decisions on technology such as whether to build an in-house solution or purchasing a third-party platform, operationalize a team, and set budgets. First the regulation must be interpreted and resources dedicated to build subject matter expertise to advise senior-level management. Then a development team must be operationalized with a data governance mandate.

Data storage costs include storing a few years worth of firm-wide recorded observable prices as well as modellability reports. Observable price data should also be made readily available by a query engine which will further increase costs to take into account data retrieval. The regulations call for using one year of observable price data in order to calculate modellability^20^ but most firms will find that extending farther back a few years will be useful for testing and auditing purposes. Additionally, the finalized reports determining the status of modellability per risk factor must be stored as well but these aggregate reports will likely not contribute a significant amount to storage costs due to its relatively compressed size in relation to the input observable price data.

The majority of long-term overall costs will likely come from the ongoing computation required to produce modellability reports. Firms may expect large variances of costs dependent on technology chosen and how the FRTB rules may change. For example, if one firm selects a cloud-based solution as opposed to an on-premise solution, the initial few years of explicit computational costs will be lower due to on-premise solutions requiring significant upfront investments in physical hardware as well as IT personnel to manage physical servers while cloud solutions can scale processing tasks on elastic machines using a subscription fee model. Cameron Fisher from *Massachusetts Institute of Technology* supports this sentiment in his paper titled “Cloud versus On-Premise Computing”:

Electing to choose Cloud is a luxury that allows the organization to avoid incurring direct internal costs such as infrastructure headcount, hardware operations, system administration, etc.^21^

Fisher also explains that elastic compute capabilities on the cloud can reduce the legacy challenge of over-provisioning or under-utilization of on-premise servers.^22^ In other words, the automated support of IT infrastructure and scaling server racks is built-in to the computational costs of cloud providers. However, the paper also explains that cloud expenditure is often measured to be higher than on-premise costs in later years after implementing a solution due to the subscription fees of computation in the cloud. Though there is a cost to managing the depreciation of on-premise physical hardware, Fisher nevertheless concludes that past a certain number of years on-premise solutions become cumulatively less costly than cloud solutions due to the ongoing cloud fees surpassing the initial investment of on-premise hardware and organizing personnel to support the physical infrastructure.^23^ The decision for implementing a cloud solution over an on-premise solution must also be made in tangent with how experts envision possible changes to the input data as well as the risk factor eligibility test itself. For example, if the volume and velocity of input data on a day-to-day basis varies significantly or there is ever a significant change to the risk factor eligibility test rules in the future, cloud solutions will be able to handle these changes more effectively by auto-scaling processing tasks while on-premise solutions may suffer from having under-utilized assets or over-provisioning issues as a result.

The computational logic and power required to run one modellability report is solely dependent on the asset class and the number of risk factors a firm wishes to calculate on. Logical operations for the risk factor eligibility test (RFET) may be broken down to 276 checks to be made per unique attribute permutation based on risk factor definition. 275 checks are required for sifting through each 90-day continuous period for 4 observable prices in the past year under part (1) of the rule. 1 check is required to query for 100 observable prices in the past year under part (2) of the rule. To give a simple example in the bond asset class, if an example firm has three different definitions of risk factors or bucketed aggregations to the data, including issue-level calculations, the data will have to be processed three times on different permutations of unique risk factor attributes. Note that different banks may have vastly different risk factor bucketing. See the chart below for the number of computational steps necessary to check for modellability on a mock sample set of: 10,000 unique bonds, 30 sectors, 22 ratings, 11 term to maturity buckets, and 50 unique issuers organized within 3 different risk factor definitions. The 22 sample number of ratings are taken from the total number of *Standard and Poor's* glossary of long-term and short-term issue credit ratings.^24^ The other sample numbers are base estimates modelled on generic issuer and sector security master metadata for Canadian bonds. The security master metadata within the bond context refers to descriptive data including sector, ratings, and issuer information associated with each price observation from ratings agencies such as *DBRS Morningstar*, *Fitch*, *Moody’s,* or *Standard and Poor's* to complement the bond prices. Note that modellability can be computed in this way only after data is already normalized, arranged, and enhanced with this security master metadata (Table 1).

**Table 1:** Number of computational checks needed given the sample set of data

Number of risk factor definitions	Risk factor bucket definition	Number of unique risk factor permutations	Checks required for modellability
3	Issue-level	10,000 unique bonds	2,760,000 checks
3	Ratings, sector, & term to maturity	7,260 permutations	2,003,760 checks
3	Issuer & term to maturity	550 permutations	151,800 checks
Total checks required for the sample risk factor definitions	4,915,560 checks		

The computational checks needed for the basic sample set provided earlier would be 4,915,560 checks per modellability report on the whole inventory of past year observable bond prices. To use IMA, the bare minimum firms are required to have modellability monitoring is on a monthly basis.^25^ In reality, most firms that decide to compute modellability will also generate extra reports mid-month so that they can have an expectation of what instruments may be modellable by month’s end. The top six Canadian banks by assets all compute modellability reports daily in anticipation for FRTB rules to take effect. Therefore, we can assume global firms will reasonably generate at least a few modellability reports per month.

Finally, the last category where a firm will incur increased technological costs when calculating modellability is in providing continuous information technology support. This includes long-term personnel to fix processes if data formats change or technologies become outdated and even the implicit costs to analyze, budget, and arrange for more server space under an on-premise implementation.

Note that there have been research in recent years to reduce additional downstream computational tasks required for IMA, for example, with Zeron Medina Laris and Ruiz’s paper that detail using the *Orthogonal Chebyshev Sliding Technique*.^26^ This method has been measured to reduce the computational burden in the calculation of expected shortfalls (ES) with different liquidity horizons or scenarios by over 90%.

## Case study: the innovative data pooling solution

As of this article’s publication, the only cloud-based FRTB data pooling solution that is being used in production is in Canada which includes the six largest banks in the country as participating members: *BMO Nesbitt Burns Inc., CIBC World Markets, National Bank Financial Inc., RBC Capital Markets, Scotia Capital*, and *TD Securities*. The solution is operated by *CanDeal Data & Analytics* service with the underlying technology built by *TickSmith Corp*, a big data financial technology provider.^27^*CanDeal* is a Canadian online exchange for debt securities jointly owned by the six largest banks. The data pool ingests real price observations from each bank, normalizes the sets, enhances the content with security master metadata, and anonymizes the private fields within the data such as the bank identifier or counterparty identifier before calculating for modellability per risk factor.

The efficacy for the Canadian data pool has been measured and published in 2019. Using the entire breadth of bond trade data from the six participating banks, data pooling increases the number of modellable instruments on an issue-level risk factor mapping for each bank on average by over 400% when compared to the modellability calculations using only each individual bank’s data.^28^ One participating bank has found an average 667% increase in issue-level modellability. In every report generated, each individual bank received at least double the number of instruments considered modellable.^29^ This is calculated by taking the same one and a half year span of historical trade data in 2018 to 2019 from each of the six banks. Processing tasks are run such that modellability reports are generated every day for half a year, as each report date requires processing the previous one year of historical data. The increase in modellability figures are calculated using only the instruments that each bank already trades within the past year. This method of measurement actually underestimates the benefits each bank receives. For example, let us take a bank that has opted to not trade a certain security in the past because of its associated capital charges. If that bank can confirm that this security is modellable due to trades from other banks in a data pool, the difference in the new lowered capital charge for trading this instrument may result in that bank deciding that trading it is now profitable. These cases of modellability increases are not included in the percentages provided. Note that specific modellability figures such as the number of unique bond issues in the set have not been publicly published for information security reasons.^30^

The Canadian data pool service with *CanDeal Data & Analytics*^31^ came into production with the six banks in January 2020. The product incorporates many elements of innovative technologies and business use-cases. It is among the first of production-grade services that stores private institutional trade data on the cloud using *AWS* and is owned by all major competitive banks in a country. Data is ingested and modellability calculations are processed using big data technologies. Data can be queried and analyzed using a massively parallel processing query engine. Information security requirements are met with each bank as data is encrypted at-rest as well as in-flight, and data is segregated in different Amazon Simple Storage Service (Amazon S3) buckets on ingest. As these storage locations are different, each participant bank still retains control and ownership of the data throughout the entire process. Data is only pooled after private fields are anonymized.^32^

**Fig. 1:**
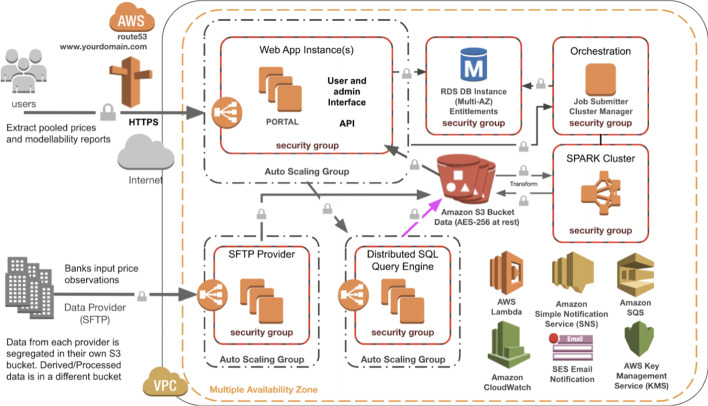
TickSmith’s example data pool architecture diagram on AWS cloud. *Note* TickSmith. "TickSmith’s Data Pooling Solution empowers Canadian banks to anonymously share data for FRTB compliance”. https://www.ticksmith.com/use-case-ticksmith-data-pooling-solution-empowers-banks-to-pool-data-for-frtb/. Retrieved 27 June 2020

*CanDeal Data & Analytics* have set FRTB working groups where all six participating banks, *CanDeal*, and *TickSmith Corp* meet bi-weekly to scope the future of the project and to discuss topics on the taxonomy, quality, and monitoring of data. These working groups provide the forum for the banks to suggest new features as well as datasets that should be onboarded for the data pool. Architecturally, the technology underlying the data pool solution has separated storage and compute machines allowing for scalability based on variable data inputs. Computational tasks are run using *Spark* on Amazon Elastic Compute instances in the cloud. See Fig. 1 below for an example architecture diagram describing the Canadian data pool:

The primary reason the data pool had been commissioned was to lower capital charges under FRTB. As of January 2020, all market risk teams of the six participating banks have been using the data pool to feed into internal risk modelling tasks in anticipation for the FRTB rules coming into effect. Since its implementation, the six Canadian banks have found a variety of additional benefits to having the data pool. These benefits include having a robust internal data distribution platform to programmatically feed consolidated data into other downstream applications according to granular entitlements such as to asset management or trade surveillance teams, the ability to create advanced evaluated pricing datasets, and for data sales by packaging country-wide data for external purchasers in collaboration with all bank participants.

Note that the data pool concept may not be applicable to other locations due to the different competition laws and economic realities of these regions. For example, Canada has fewer banks and less fragmentation in the banking sector than the United States, as a result it is easier in Canada to gather a group of banks that have mutual interests in proving the historical prices of similar securities. Additionally, the number of unique Canadian dollar debt securities traded by the top six Canadian banks remain lower and more homogenized than US debt securities which are among the most diverse and liquid in the world.^33^ The Canadian banking sector may be special in the way it is considered a “monopolistically competitive industry” according to Jason Allen and Walter Engert within *Bank of Canada’s* Department of Monetary and Financial Analysis^34^ that is also able to facilitate this collaboration between banks; though the Nordic region has also been identified as a likely candidate for an FRTB data pooling implementation by *Risk*.^35^

## Data pooling: limitations and evaluation

Data pooling with the six Canadian bank participants has been shown to facilitate the increase of modellable risk factors for the bond asset class consistently by several factors with average increases across all banks by over 400%.^36^ However, a limitation with this study is that granular trading information, even aggregate positions the banks may hold, cannot be published as they are considered private institutional metrics. Additionally, use of country-wide governmental data, such as the IIROC Rule 2800C bond trade set in Canada, is not helpful in the context of improving modellability from a bank consortium’s perspective. This paper’s examination of modellability using the bond asset class from the bank participants targets both interest rate and credit spread risk types. As stated before, the increased modellablity each participant achieves when pooling data allows them to use the less capital-intensive IMA method for a wider breadth of instruments to value risk resulting in considerable savings in capital costs for all participants. “Group 1” banks that responded to the April 2020 *BCBS Quantitative Impact Study (QIS)* have stated that the mean capital requirements using the STA sensitivities-based method is expected to be 35.2% whereas the mean capital requirements using IMA is 19.6%. However, there are a few additional key items to consider with the data pooling solution. Participating banks must ensure their information security requirements are met and private data is encrypted at all stages from the ingestion process to the anonymization and modellability report generation steps. Participants should also ensure that the operator of the data pool is a third-party so no conflicts of interests arise and the underlying technology is secure with granular entitlements for both internal users and outside entities. Private data should be received in segregated environments and private attributes should be redacted and anonymized when the data is pooled so sensitive data never coexists in the actual pool.

Another element to consider is that participating banks may contribute different levels in terms of breadth as well as depth of trading data to the pool. This may create a situation where a participating bank that contributes more valuable data to the pool benefits, in relative terms, less than a bank that contributes a lesser amount to the pool. The incentives for contributing here are misaligned as smaller banks with less to contribute may have more to gain from the pool. Therefore, the operator of the data pool should analyze contributed data to determine how to remunerate higher contributing banks or limit the gains taken by lower contributing banks. These drawbacks may also impact the feasibility assessment of a region considering the implementation of a data pool solution.

Macro factors that may affect the feasibility of implementing a data pooling solution includes the competition laws in the region, how fragmented the region’s banking sector is, and the homogeneity of traded instruments among the participants. In fact, a significant risk can come from the fact that there are certain instruments that only a few participants trade but not others. By allowing these instruments into the pool, the anonymization feature of the data pool may be partly compromised as participants can guess at which other banks are trading these instruments and obtain the prices as well as dates these instruments are traded on. There is a difficult balance to achieve here as the data pool specifically targets the problem of making illiquid instruments modellable. It is to be noted that because of this reason, the more participants there are within data pooling, the more effective and anonymized the pooled data is. However, as more participants enter the pool, it becomes more difficult for the data pool’s operator to assess the value of each contributor’s data to maintain a fair data pooling product.

## Conclusion

Each firm must evaluate whether the lower capital charge fees they will incur by using IMA justifies building a comprehensive data platform. Regardless, a firm modernizing their data operations and technologies will have far-reaching benefits outside of just lowering capital charge fees for FRTB. As data technologies advance and scalable data storage as well as computation becomes more affordable, regulations are heading in the direction of requiring more granular data points with more stringent retention requirements.

Basel III FRTB set of rules in particular require significant investment to implement business logic, build data storage components, computation components, and continuous information technology support. This investment may be costly but is in line with making data systems more robust in the financial world and can open other avenues of innovation such as data pooling initiatives to further lower capital charge fees. The global financial regulatory landscape is changing with big data technologies and firms will have to innovate their data systems not just to outperform competitors but to comply with regulations and keep business as usual.

Data pooling in Canada was implemented with a mandate to modernize and consolidate data from legacy systems across different banks. The data pool has proven to be beneficial to all bank participants for market risk, data monetization, and data distribution use-cases. The Canadian data pool serves to provide comprehensive pooled prices and modellability reports to lower capital charges once the FRTB rules fall into place in 2023.

## Data Availability

Data and material are available in public databases. See the references section and footnotes for all material used.

## Footnotes

1 CanDeal. “CanDeal partners with six Canadian Banks to Create Market Data Hub”. http://www.candeal.ca/news/news-press-releases/2019-02-12/candeal-partners-six-cana dian-banks-create-market-data-hub. Retrieved 3 February 2020.

2 Barnett-Hart, Anna Katherine. "The Story of the CDO Market Meltdown: An Empirical Analysis" (PDF). *March 19, 2009*. Harvard Kennedy School. Retrieved 3 February 2020.

3 The Financial Crisis Inquiry Commission “Final Report of the National Commission on the Causes of the Financial and Economic Crisis in the United States”. Page XXV. *THE FINANCIAL CRISIS INQUIRY REPORT*. 25 February 2011, www.govinfo.gov/content/pkg/GPO-FCIC/pdf/GPO-FCIC.pdf. Retrieved 3 February 2020.

4 The Financial Crisis Inquiry Commission “Final Report of the National Commission on the Causes of the Financial and Economic Crisis in the United States”. Page 3–4. *THE FINANCIAL CRISIS INQUIRY REPORT*. 25 February 2011, www.govinfo.gov/content/pkg/GPO-FCIC/pdf/GPO-FCIC.pdf. Retrieved 3 February 2020.

5 Bank for International Settlements. "Sixteenth progress report on adoption of the Basel regulatory framework". Page 1. https://www.bis.org/bcbs/publ/d464.pdf. Retrieved 3 February 2020.

6 Bank for International Settlements. "Sixteenth progress report on adoption of the Basel regulatory framework". Page 1. https://www.bis.org/bcbs/publ/d464.pdf. Retrieved 3 February 2020.

7 Barnett-Hart, Anna Katherine. "The Story of the CDO Market Meltdown: An Empirical Analysis" (PDF). *March 19, 2009*. Harvard Kennedy School. Retrieved 3 February 2020.

8 Bank for International Settlements. "Minimum capital requirements for market risk". Page 1. https://www.bis.org/bcbs/publ/d457.pdf. Retrieved 3 February 2020.

9 Bank for International Settlements. "Governors and Heads of Supervision announce deferral of Basel III implementation to increase operational capacity of banks and supervisors to respond to Covid-19". https://www.bis.org/press/p200327.htm. Retrieved 13 May 2020.

10 Bank for International Settlements. "Minimum capital requirements for market risk". Page 54–56. https://www.bis.org/bcbs/publ/d457.pdf. Retrieved 3 February 2020.

11 European Central Bank. “Market risk: implementing new rules for internal models”. https://www.bankingsupervision.europa.eu/press/publications/newsletter/2020/html/ssm.nl200212_2.en.html. Retrieved 20 April 2021.

12 Bank for International Settlements. "Minimum capital requirements for market risk". https://www.bis.org/bcbs/publ/d457.pdf. Retrieved 3 February 2020.

13 Bank for International Settlements. “Basel III Monitoring Report”. Page 165. April 2020, https://www.bis.org/bcbs/publ/d500.pdf. Retrieved 19 May 2020.

14 Bank for International Settlements. “Basel III Monitoring Report”. Page 165. April 2020, https://www.bis.org/bcbs/publ/d500.pdf. Retrieved 19 May 2020.

15 Bank for International Settlements. "Minimum capital requirements for market risk". Page 74–75. https://www.bis.org/bcbs/publ/d457.pdf. Retrieved 3 February 2020.

16 Zhan (2020).

17 Zeron Medina Laris and Ruiz (2019).

18 JPMorgan Chase & Co. “1Q19 FINANCIAL RESULTS”. https://www.jpmorganchase.com/corporate/investor-relations/document/1Q19-earnings-transcript.pdf. Retrieved 20 May 2020.

19 CNBC. “HSBC plans more China tech jobs in push for market share”. https://www.cnbc.com/2019/05/21/hsbc-plans-more-china-tech-jobs-in-push-for-market-share.html. Retrieved 20 May 2020.

20 Bank for International Settlements. "Minimum capital requirements for market risk". https://www.bis.org/bcbs/publ/d457.pdf. Retrieved 3 February 2020.

21 Fisher (2018).

22 Fisher (2018).

23 Fisher (2018).

24 S&P Global Ratings. “S&P Global Ratings Definitions”. https://www.standardandpoors.com/en_US/delegate/getPDF?articleId=2017758&type=COMMENTS&subType=REGULATORY. Retrieved 10 May 2020.

25 Bank for International Settlements. "Minimum capital requirements for market risk". Page 74. https://www.bis.org/bcbs/publ/d457.pdf. Retrieved 3 February 2020.

26 Zeron Medina Laris and Ruiz (2019).

27 Bowie, Max. "CanDeal Enlists TickSmith for OTC Canadian Data Pool Tech". Waters. https://www.waterstechnology.com/data-management/4202926/candeal-enlists-ticksmith-for-otc-canadian-data-pool-tech. Retrieved 3 February 2020.

28 Nicholson, Richard & Schmidt-Tank, Stephan. “How cloud increases flexibility of trading risk infrastructure for FRTB compliance”. Amazon Web Services. https://aws.amazon.com/blogs/industries/how-cloud-increases-flexibility-of-trading-risk-infrastructure-for-frtb-compliance/. Retrieved 10 August 2020.

29 Amazon Web Services. “AWS Case Study: CanDeal Uses TickSmith to Help Address FRTB Compliance”. https://d1.awsstatic.com/Solution%20Space%20(CRS)/AWS-Case-Study-FRTB-AWS-05202020-Final.pdf. Retrieved 27 May 2020.

30 TickSmith. "Finally, a risk solution for fundamental review of the trading book (FRTB) regulations”. https://www.ticksmith.com/a-risk-solution-for-fundamental-review-of-the-trading-book-regulations/. Retrieved 3 February 2020.

31 CanDeal. “CanDeal partners with six Canadian Banks to Create Market Data Hub”. http://www.candeal.ca/news/news-press-releases/2019-02-12/candeal-partners-six-canadian-banks-create-market-data-hub. Retrieved 3 February 2020.

32 Amazon Web Services. “AWS Case Study: CanDeal Uses TickSmith to Help Address FRTB Compliance”. https://d1.awsstatic.com/Solution%20Space%20(CRS)/AWS-Case-Study-FRTB-AWS-05202020-Final.pdf. Retrieved 27 May 2020.

33 Allen, Jason & Engert, Walter. “Efficiency and Competition in Canadian Banking”. Bank of Canada Review. 2007.

34 Allen and Engert (2007).

35 DeFrancesco, Dan. "FRTB: Nordic banks mull regional data pool". Risk. https://www.risk.net/risk-management/5593441/frtb-nordic-banks-mull-regional-data-pool. Retrieved 3 February 2020.

36 Amazon Web Services. “AWS Case Study: CanDeal Uses TickSmith to Help Address FRTB Compliance”. https://d1.awsstatic.com/Solution%20Space%20(CRS)/AWS-Case-Study-FRTB-AWS-05202020-Final.pdf. Retrieved 27 May 2020.
